# Gnosis and counterstories: decolonial disability reflections on delinking as a transgressive social methodology

**DOI:** 10.3389/fsoc.2025.1578464

**Published:** 2025-07-18

**Authors:** Alexis Padilla, Paulo Tan

**Affiliations:** ^1^School of Disability Studies, Faculty of Community Services, Toronto Metropolitan University, Toronto, ON, Canada; ^2^Education Department, University of California, Santa Cruz, Santa Cruz, CA, United States

**Keywords:** counterstories, disability, epistemology, mathematics education, decolonization

## Abstract

This essay articulates an innovative counterstory-based methodology of decolonialde linking which disrupts the very epistemic foundations of sociological disciplinary boundaries and ways of thinking about the production and distribution of knowledges. As non-white co-authors, we have opted to follow to adopt an expansive conception of decolonial/border-thinking gnosis and delinking as a way to embrace all knowledges, particularly those which do not conform to disciplinary modes of exposition and rationalist systematicity within the epistemic conceptions of knowledge. Using two disabled counterstories as gnosis illustrations, our essay shows how their enactment transgresses established norms for addressing and engaging with traditional, discipline-bound epistemological concerns. As such, we aim to open theoretical and methodological avenues for decolonial and non-Eurocentric spheres of imagination. More specifically, since the worlds of mathematics and mathematics education are so dominated by rationalist and neurotypical epistemologies grounded on the Cartesian duality of matter vs. ideas, both of our illustrative counterstories will deal with aspects that disrupt such epistemological paradigms through intersectional cripistemologies.

This essay articulates an innovative counterstory-based methodology of decolonial delinking which disrupts the very epistemic foundations of sociological disciplinary boundaries^1^ and ways of thinking about the production and distribution of knowledges. Former American Sociological Association President Aldon Morris (2015) has already questioned the way racialized conceptions of modernity during the era of sociology's birth ended up marginalizing the scholarly status of non-white classical sociologists such as W. E. B. Du Bois, the founder of the first sociological school in the United States. As non-white co-authors Alexis identifies as Latinx and disabled; Paulo identifies as Chinese-American and disability advocate and caretaker. As such, we have opted to follow Walter Mignolo's (2000, 2007, 2021) radical suggestion to adopt an expansive conception of decolonial/border-thinking gnosis and delinking^2^ as a way to embrace all knowledges, particularly those which do not conform to disciplinary modes of exposition and rationalist systematicity within the epistemic conceptions of knowledge.

As Mignolo (2000) explains in the introduction to his volume on border-thinking, both gnosis and episteme are used to translate the word knowledge from the Greek. However, only the former captures non-systematic modalities of knowledge, encompassing all forms of wisdom seeking processes and activities. Thus, gnosis enables us to tap into nomad and non-colonized knowledges in line with the broad non-rationalist thinking space that Deleuze and Guattari (1987) encompass under the non-hierarchical analytical term nomadology. Within epistemic conceptions of existing sociological methodologies, the emphasis is placed on formal disciplinary systematicity in line with Eurocentric modernity principles. Not surprisingly, therefore, classical sociology only recognizes European “fathers” of the discipline, e.g., Marx, Weber, Durkheim and so forth. This in turn excludes multiple forms of non-rational and para-rational wisdom seeking mechanisms located within the gnosis decolonial and border-thinking spaces that Mignolo and other Latin American philosophers call radical exteriority (Vallega, 2014). Worst still, this Eurocentric epistemic tendency deliberately suppresses critical examinations of what Peruvian sociologist Aníbal Quijano (1991, 2000, 2007) characterizes as the intrinsic structural and ideological interdependence between modernity, rationality and coloniality.^3^ Being a sociologist by training, Quijano himself exemplifies the enactment of gnosis vs. epistemology by not circumscribing his work to the disciplinary confines of sociology. Quijano radically engages with various emancipatory knowledges and currents of wisdom, what has evolved into the dynamic sub-fields of decolonial and anti-colonial thought (Mignolo, 2021, preface).^4^ Mignolo thus favors the philosophical term gnoseology^5^ which encompasses the study of all knowledges, and which was in use during scholasticism, that is prior to modernity.

Using two disabled LatDisCrit and pan-Asian DisCrit^6^ counterstories (Padilla, 2021) as gnosis illustrations, our essay shows how their enactment transgresses established norms for addressing and engaging with traditional, discipline-bound epistemological concerns. As such, we aim to open theoretical and methodological avenues for decolonial and non-Eurocentric spheres of imagination. More specifically, since the worlds of mathematics and mathematics education are so dominated by rationalist and neurotypical epistemologies grounded on the Cartesian duality of matter vs. ideas, both of our illustrative counterstories will deal with aspects that disrupt such epistemological paradigms through intersectional cripistemologies (Johnson and McRuer, 2014; Sandahl, 2003). These intersectional cripistemologies are unique insofar as they lean instead toward the enactment of decolonial gnosis. In so doing, we open delinking spaces of alternative anti-colonial imaginaries; thus, we pursue decolonial disability justice and anti-supremacist paradigms that expand social movement and public sociology's horizons. Moreover, we examine the impact that these explorations have in advancing equity concerns in mathematics and mathematics education via the transgression of their Eurocentric, colonial and neo-colonial foundations (Andrade-Molina and Valero, 2015, 2017). In the following section, we start by explaining the practical gnosis meaning of delinking in relation to dewesternization and decolonial processes which, while being parallel and aimed at disrupting colonizing and westernizing knowledge, power, ways of being and value systems, are not identical in their scope and agentic implications. Next, we explore the epistemological and gnosis meaning of counterstories as counternarrative social imagination tools, introducing and developing our two illustrative counterstories. Our essay concludes by exploring the emancipatory possibilities of gnosis and delinking in the realm of decolonial public sociology. We give preeminence to intersectional cripistemology spaces as the tip of the iceberg in an era marked by recalcitrant moves away from inclusive equity and cognitive respect to different ways of knowing, being and becoming.

## From gnosis to delinking to decoloniality enactments

In this section, we address the nexus between gnosis, delinking and decoloniality. In addition to what we said in footnote 2 about delinking as a movement away from discipline-bound epistemological systematicity, it is important to establish several key dimensions associated with delinking as an innovative notion tied to decolonial cross-coalitional movement building as we use it in this essay. First, we stress that the gnosis-driven movement toward delinking is not merely or even mainly concerned with what Mignolo (2021) calls dewesternization. Mignolo points out emphatically that the process of dewesternization is one of the parallel dynamic historical trends that have resulted from the implosion of westernization's end of era which covered roughly from 1500 to 2000. Being the first era truly global in nature (Quijano and Wallerstein, 1992; Wallerstein, 1974), the westernization era was characterized by “political and economic unilaterality, and epistemic and aesthetic universality” (Mignolo, 2021, p. x). As a result of its implosive demise, cognitive and sociopolitical changes which before were regarded as unlikely, are becoming not only possible but tangible under new global realignments. “The pandemic only accelerated a process that is irreversible and provided more evidence that the long-lasting consequences of coloniality are no longer hidden under the rhetoric of modernity, development, progress, growth, ‘more is better,' and ‘bigger is virtuous”' (Mignolo, 2021). Secondly, in terms of our unique delinking emphasis, unlike the gnosis-driven power that results from conceptualizing delinking as border thinking spaces of decoloniality in action, the trajectory toward dewesternization has also been unilateral and strategically imposed by non-western forces driven by nation state agendas. Despite establishing the end of westernization as an era around the year 2000, Mignolo also recognizes that dewesternization had already been strategically promoted, especially in the last seven decades or so through state action which initially came about during the anti-colonial movements that proliferated in Africa and some Caribbean nations between the 1950s and the 1970s, but now it operates primarily through the China, Iran, Russia axis (Mignolo, 2021, p. 17 and following). The core decolonizing event in Mignolo's chronology is the Bandung Conference hosted by Indonesia in 1955. We do not have space to analyze in depth the dynamics this conference unleashed, especially regarding antiracist and non-aligned geopolitical movements (see, Mignolo, 2021, ch. 9). However, the conference represents a crucial geopolitical differentiating component to understand our third point when it comes to the unique kind of delinking we are underscoring, which is that decolonization and decoloniality are fundamentally different in terms of inter-state relations. Decolonization is about the undoing of various modalities of colonialism which operate through inter-state relations. Decoloniality is much broader; it tackles the analysis and dismantling of the colonial matrix of power (CMP).^7^ Mignolo adds that CMP's operational elements have undergone three crucial processes that are by no means linear, often coexisting in complex ways since the year 1500: constitution, destitution and restitution. Coloniality and westernization were responsible for the first two of these processes. Yet, when it comes to restitution, both decoloniality and dewesternization are at work in parallel through very different mechanisms. Technically, therefore, both of them involve some form of delinking. Nonetheless, as we explain below, the operationalization of decolonial delinking as a distinctive sphere gets enacted within political society. In other words, it is reserved to dynamics that take place outside state spheres. This makes decolonial delinking particularly capacious for propelling border-thinking spaces through gnosis. In turn, its reconstitution is truly revolutionary, elevating all knowledges and giving peoplehood true intersectional and cross-coalitional agency. This gnosis-based kind of delinking thus operates in ways that radical solidarity becomes possible and genuinely transformational, activating so far marginalized wisdom seeking endeavors with actionable transgressive consequences (Padilla, 2018; Gaztambide-Fernández, 2012).

Our argument therefore in positioning delinking as intrinsic to gnosis-centered collective action, along with Mignolo's (2021) framing of decoloniality as an emancipatory ethos, is careful to stress that, ultimately, the decolonial project must not be a state sponsored process. Rather, it needs to be the product of agentic and collective enactments born within the dynamic sphere that Mignolo, following Chatterjee (2011) calls political society. It is interesting that Mignolo opts not to adopt the Marxian/Gramscian civil society terminology which has much more diffused usage in sociological circles. The reason stems from the desire to accentuate the political nature of decoloniality along with the kind of explicit sociopolitical orientation of the collective action involved in its brewing (see, e.g., Kumar Patnaik, 2025). To be sure, not all resistance collective action is decolonial; and as will be seen, the distinguishing features of decolonial collective action have to do with gnosis and delinking. In other words, as Fregoso Bailón et al. (2024) make clear, it is not enough to call something decolonial and still remain within the confines of western epistemological rationalism. To “overcome the present theoretical indifference, it is essential to detach ourselves from the colonial matrix—the oppressive logic that underpins Western society—and adopt a nomadic perspective that seamlessly transitions between diverse epistemological frameworks” (Fregoso Bailón et al., 2024, p. 2; see also, Paraskeva, 2022, p. 353 and following). Along with Fregoso et al., we are convinced that the nomadic and itinerant are intrinsic to gnosis-based knowledges and collective endeavors since they are not tied to disciplinary and rigidified rationalist boundaries. This is why Mignolo's conceptualization of border thinking adopts an itinerant stance. Border thinking deliberately adopts a suspicious attitude toward Eurocentric epistemologies and thus learns to delink from them. As Mignolo (2000, n.p.) himself puts it, border “thinking (or “border gnosis” …) is a logical consequence of the colonial difference. It can be traced back to the initial moment of Spanish colonialism in the Andes and Mesoamerica.” That was the precise moment when Eurocentrism gave birth to its hierarchical ethos: when Europeans realized they could rule over previously unknown territories and racial categories. The Europeans started the complex process of self-shaping and “modernizing” via colonial extraction and redefining racialized civilizational walls under which disability also started emerging as a distinctively subaltern space, even for disabled Europeans. As such, the sense of cognitive dissonance of subaltern categories, disability included, traces back to what Wallerstein (1974) designates as the long sixteenth century, the period when world-system economic and sociopolitical structures and relations came into play. This dissonance to which Mignolo alludes stems from the hierarchical categorization of knowledges engendered by coloniality and modernity working in tandem. Their interlocking supremacist oppression mechanisms of exploitation and cultural/racializing/ableist marginalization work hand-in-hand with the placement of rationality above all forms of thinking and knowing. This happened despite many kinds of gnosis having existed and developed in parallel throughout the world without the global hindrance of the kind inaugurated by Eurocentrism (Alcoff, 2021), mathematics and mathematics education, of course, being among the disciplines where this tendency is most overwhelming (Andrade-Molina and Valero, 2015, 2017).

In terms of philosophy, for example, Mignolo points to Mudimbe's (1988) *The Invention of Africa: Gnosis, Philosophy and the Order of Knowledge* as one of the first volumes that made evident the need to abandon purely epistemological treatments of rationalist knowledge in order to embrace knowledges which do not conform to its reductionist parameters of thinking and understanding. However, it is paramount to look deeper at the sociopolitical dimensions of decoloniality as a freedom-seeking process. To this end, it is worth pausing briefly to understand better what Chatterjee (2011) means by political society as opposed to what European Marxian thinkers like Gramsci call civil society.

Chatterjee (2011) points out that there are classical concepts of political theory such as family, civil society, political society, and the state which nonetheless tend to be used most inconsistently. Hegel, for instance, elevated the family, civil society and state, but did not have a place for political society in his institutional modeling of sociopolitical arrangements (Hegel's choice is important because he influenced Marx and a good number of subsequent thinkers e.g., Gramsci). By political society Chatterjee means the domain of institutions that mediate between people and the state but which stand in parallel to civil society. Civil society is restricted to more or less autonomous voluntary associations (e.g., churches and non-profits) without explicit sociopolitical missions, very much in line with what Putnam (2000) examines as social capital networks of trust. In postcolonial societies, these civil society networks of trust are often coopted by the state through clientelist mechanisms. This, in turn, forces spheres of resistance and change making to reside within that alternative sphere of political society to which Chatterjee gives preeminence. It is also in this context of resistance and emancipatory spaces where it makes most sense to excavate the dynamic ethos of counterstories as incubation domains of gnosis in action. This is what we foreground in the section that follows. Importantly, as an explicit delinking move, readers should note that the kind of embodied counterstory we have in mind and develop later in our essay, does not attempt to purify pedagogical mathematics practices to make them cosmetically liberatory insofar as they become somehow less ableist. Our gnosis-based aim is more radical. We want to tap into non-disciplined or undisciplined modalities of so-called minor mathematics (de Freitas and Sinclair, 2020). We do so on purpose to show that, especially within the domain of so-called profound and severe disabilities (and all intersectional disability spaces and experiences for that matter), there is powerful mathematical brilliance. In other words, we want to push against the famous Greek root of pedagogy as literally meaning the holding children by the hand to guide them somewhere (particularly since that somewhere is presumed not to be under the control of the child/learner). In terms of gnosis-based intersectional decoloniality, what is needed is exactly the opposite. We need to let go of their hands, bodies, dreams and souls so as to facilitate the free expression of these brilliances. Furthermore, we need to honor them as valuable and revolutionary funds of knowledge and anti-epistemological, that is, anti-disciplining modalities of delinking. We do so through the telling of their counterstories but there are probably multiple ways to achieve this aim and elevate such amazing myriad manifestations of subversive creativity.

## Decolonial delinking through counterstories

We start this section by elevating the gnosis power of embodied, first person experiential counterstories whose ethos typically operates at the micro-level of analysis. Notwithstanding this micro-relational emphasis, embodied counterstories, especially in their intersectional anti-ableist contours are intrinsically political. They are not mere isolated vignettes. They do not give voice to a choir of depoliticized subjects. Herein thus resides their decolonial and situated emancipation gnosis-based spirit. Decolonial delinking through the power of counterstories, therefore, involves a deliberate process of anchoring collective action on gnosis as opposed to epistemology. Dimensions like poetic resistance, counterstory telling and non-rationalist wisdom seeking mechanisms acquire centerstage. The border-crossing work pursued by Chicana feminist thinkers such as Gloria Anzaldua comes to mind here. Look for instance at how Anzaldúa (2015, n.p.) frames her gnosis-oriented knowledge pursuits:

Leaving home has cast you adrift in the liminal space between home and school, bereft of your former frame of reference. In class you feel you're on a rack, body prone across the equator between the diverse notions and nations that compose you. Remolinos (whirlwinds) sweep you off your feet, pulling you here and there. While home, family, and ethnic culture tug you back to the tribe, to the chicana indígena you were before, the anglo world sucks you toward an assimilated, homogenized, whitewashed identity. Each separate reality and its belief system vies with others to convert you to its worldview. Each exhorts you to turn your back on other interpretations, other tribes. You face divisions within your cultures—divisions of class, gender, sexuality, nationality, and ethnicity. You face both entrenched institutions and the oppositional movements of working-class women, people of color, and queers. Pulled between opposing realities, you feel torn between “white” ways and Mexican ways, between Chicano nationalists and conservative Hispanics. Suspended between traditional values and feminist ideas, you don't know whether to assimilate, separate, or isolate.

It is interesting that, although the word disability is never used by Anzaldua, a lot of what she describes here seems in line with what mad activists (see, e.g., Price, 2011) would characterize as part of their everyday sense of non-rationalist reality embracing processes and their sense of pluriversal political ontology (to use Arturo Escobar's, 2020 terminology). Furthermore, there is an obvious intersectional, rather fragile quest for finding mechanisms of resistance. In many ways, our respective counterstories below mirror several of these characteristics. In particular, these counterstories' focal agentic struggles operate within the broader confines of mathematics, a discipline that epitomizes the rationalist power of Eurocentric epistemologies as well as ableist, gendered and racialized ideologies of western knowledge supremacy (Mikulan and Sinclair, 2024).

## Paulo and Kai's counterstory: in search of gnosis as a catalyzing liberatory space

Profound is a word used by non-disabled folks (and unfortunately many disabled individuals as well) to describe a category of disability that warrants certain types of responses, interventions, practices, policies, etc. in US K-12 schools. It's often coupled with the word severe. The category severe-profound disabilities signal an almost nullified educational expectation where so called life-skills take precedence over academic ones (Jorgensen et al., 2010). Simultaneously, it applies to a particular level of support that, although qualified as needed for those who are so labeled and thus determined to fall within this category, is also regarded as not fundamentally useful to push the learning limits of a space so mysterious that ends up in practice deemed as pedagogically pointless and devoid of false achievement hopes. Professionals such as educators and psychologists had determined that Kai (pseudonym) who is now 21 years-old and someone who I have advocated for, fell into the severe-profound disability category. This happened as his first encounter with the public education system in the US at the early age of three. As with Kai, those identified in the severe-profound category are most likely to be segregated from their non-disabled peers (i.e., taught in separate classrooms and/or schools) and engage in an extremely watered-down curriculum for the entirety of their K-12 schooling experience. What's fascinating is that the word profound is also used in other, more valuable senses. It is used to describe the depth of knowledge and learning that one experiences over a long-course period. As a Chinese-American advocate and caretaker for and with persons with profound disability, mathematics education scholar, brother, son, immigrant, and non-disabled person, I'd describe my experiences with advocating for Kai and his parents as a profound form of knowledge and emotional production and distribution that has evolved over the past 21 years. This evolution and complex sense of becoming has involved enriching, ongoing, and entangled experiences with them which have transformed my own sense of being and my own capacity for non-rationalist wisdom cultivation.

### Early interventions

Kai was diagnosed with autism at the age of two. During that time I was just starting my doctoral studies in mathematics education. The psychologist who made the diagnosis suggested to Kai's parents to “drop everything” and focus on intensive levels of early interventions to ensure Kai the best possible outcomes in terms of reducing the problems associated with autism. That is to say, these interventions aimed at fixing autism to make Kai function more normally. So addressing autism early was crucial to ensure a more normal human development. At that period in my life, I fully agreed with the prospects of having Kai become more normal. Like me and his parents Kai is an East-Asian appearing person. Relatively speaking, autism at the time was still relatively new and was framed in very tragic and scary ways. A team of white-female educators developed an Individualized Family Service Plan (IFSP) for Kai. The IFSP is a legally binding document that specifies which services and supports the state will provide for young children. IFSPs stipulate in-home services such as occupational, speech, and play therapy with Kai and his parents as participants. The focus of these therapies was not only to remediate areas were Kai lagged his typically developing peers but also to train parents to provide these therapies. These therapies were and continued to be underpinned by Western cultural norms (e.g., taking turns, individualistic, making eye contact).

Kai's parents shared with me that they really dreaded these therapy sessions. They had a hard time focusing and would often find themselves dozing off. More concerning was that Kai would start crying once he associated the ring of the doorbell with the therapist's arrival to conduct a session. The sessions focused on therapist modeling interventions strategies on Kai so that his parents could implement them regularly throughout Kai's day. These interventions were guided by applied behavior analysis (ABA) techniques such that desired behaviors (e.g., making eye contact) could be shaped while undesired behaviors (e.g., stimming) would be extinguished or replaced with a more appropriate behavior. Scholars have critiqued ABA as a dehumanizing approach used to normalize autistic folks (e.g., Broderick, 2009; Roscigno, 2019; Williams, 2018). Kai did and continues to stim regularly. At the time, his parents and I considered his stimming a big problem as it interfered with his therapy. His parents demanded him to quiet his hands, to sit still, and make regular eye contact as a way to have him more focus on learning activities to meet certain able-bodied learning goals and Westernized norms. His parents noticed that Kai tried, but never to an acceptable level as his stimming needs would usually be too overwhelming. Of course, reframed as expressivity, these needs acquire such a different aura. This is an aura that when thinking of disciplinary rationalist spaces such as those of mathematics reflect a powerful anti-disciplinary, gnosis-based delinking revolution whose very transgressiveness opens up the door to funds of knowledge as of yet unexplored in traditional, deficit-centered pedagogical spaces (de Freitas and Sinclair, 2014).

During this time, I did more self-learning on ABA and I became fascinated by some of the prominent research in this area. In particular, I read about the University of California, Los Angeles (UCLA) Young Autism Project and was amazed by the results the researchers were able to achieve with intense-levels of ABA therapy with young autistic children. This research showed that children who received these interventions were indistinguishable from normally developing children which I now realize showed clearly their ableist bias by centering on enacting normalcy ideology and practices. As his advocate, these were the results I all wanted for Kai, to be normal. It's paramount to realize that this desire for normalcy is an assimilationist stance that many immigrants to the US, such as Kai's parents and I, who strive to just fit in. We wanted Kai to have everything that we wanted in life, the so-called American dream, buying a house, having a family, and having a good paying job. ABA appeared to provide Kai and his parents with a key to that dream. What's wrong with that?

### Rejecting systems of oppressions, unlearning hegemonic knowledges

Between those early years in Kai's life and now, Kai and I have evolved and continue to do so in profound and radical ways—an earthquake of knowledge development. Our on-going process involving co-becoming, struggling, studying, and the experiences navigating the oppressive environments that we co-inhabit has resulted in our current resistance stance, one that I describe as anti-ableist, antiracist, and anti-colonial (in this case meaning more than decolonial at the micro-level of resistance, see, e.g., Scribano, 2021).

As I developed my own critical consciousness since resuming my doctoral studies, I began to notice Kai's agency and unique gnosis-based expressivity as a disabled person of Chinese descent. I now realize that his agency unfolds through acts of resistance against a colonizing society not built for disabled folks (Ho, 2020) and their intersectionalities (Piepzna-Samarasinha, 2018). Thus, a critical theory, reflection, and praxis (Freire, 1970/2000) relationality occurred and continues to occur within and between Kai and me and beyond. Our daily interactions have shaped and keep being shaped by what we each bring. Kai brings his set of theories in the flesh (Moraga and Anzaldúa, 1981), one based on his lived experiences as an autistic and Chinese-American male who uses non-speaking modes of communication. I bring my experience as an academic who studies advancing intersectional disability justice in and through mathematics education. I also spent 10 years as a public school mathematics teacher in the US. In turn, my own experiential growth as educator and scholar has made me realize that the knowledge that develops from our interactions is profound in that it is a culmination of particular forms of relationalities, experiences, and theories that focus on and transcend mathematics education.

In particular, I've gained profound levels of understanding of disabilities and have applied this understanding to my personal and professional lives. For me, these two spheres of life are not separate but are intimately entangled. For example, throughout Kai's K-12 schooling experiences, I was involved as a member of his Individualized Educational Program (IEP) meetings whereby my knowledge informed and was informed by those often contentious encounters. Coupled with my own set of experiences and expertise, I attempted to bring Kai's unique gnosis-based ways of knowing and doing mathematics from outside of schooling contexts to inform his IEP goals in mathematics—a form of knowing and doing that counters but also expands dominant forms of mathematics education or what de Freitas and Sinclair (2020) call “major mathematics.” I wanted these goals to depart from building on gnosis dimensions instead of relying on dominant epistemic ways of knowing and educating which, as should be evident to the reader, are charged with ableist, normalcy-imposing assumptions. For example, most educators ascribe to ideals that verbal and written forms of mathematics expressions are the only ways a student can demonstrate mathematics learning and competency. Here IEPs heavily reinforce these ideals with the requirement that goals need to be observable and measurable which taken at the surface level can be severely restrictive. Thus, I proposed more transgressive IEP mathematics goals (e.g., moving from only narrow skills-based goals to ones focused on communicating mathematical reasoning^8^) as well as the supports needed to meet these goals (e.g., teacher professional development and resources) that directly challenge these ideals while affirming more expansive ways of knowing and doing mathematics (e.g., delinking measurability and quantification). In doing so, I recognize this work as anti-ableist, antiracist, and anti-colonial in its delinking ethos. This work is also profound for many mathematics educators in recognizing a much broader realm of mathematics knowing and doing that exist in their classrooms. Thus, for both Kai and myself, the resistance work at stake is about de-centering dominant forms of mathematics education that have privileged and continue to elevate Eurocentric, able-bodied, and colonizer mathematics while profoundly enriching this relational field.

## Alexis' counterstory: on the crafting and abandonment of improvisational gnosis spatiality of minor mathematics

In this second counterstory, we take up once more the theme of intersectional decoloniality through gnosis-based delinking. Yet in this case, the framing comes through the embodied experiences of a brown blind male working-class agent. As a disabled person residing in the global South, I was faced with a critical tension regarding my love for music, which in my case, meant an intimate engagement with what de Freitas and Sinclair (2020) encompass under the expression minor mathematics, those that “are often erased by state-sanctioned curricular images of mathematics” and “buried under ‘major' settler mathematics” (p. 1). In the world of science education, it is relatively common to hear the expression citizen science (c.f., Herodotou et al., 2018). Nonetheless, minor mathematics goes further by underscoring gnosis-centered ways of thinking and doing mathematics completely outside of the discipline and especially out of classroom-bound ecologies of learning. I fit this profile since I never was what you could call a professional mathematics or mathematics education practitioner, as was clearly the case with Paulo. For me, that engagement came vicariously, especially as I studied musical theory and musical notation during my conservatory years, learning to master the flute in the 1980s. Those studies mimicked the oppositional tension between gnosis and episteme. By the time I went to the conservatory I had already mastered the recorder. However, in the case of the recorder, my engagement reflected a fluid, much less formal regime which in my home country was alluded to among musicians under the informal, almost disrespectful nomenclature of “guataca.” As a radical gnosis, rather undisciplined experience of learning and performing the embedded minor mathematics of music, guataca is a delinking term. It gets reserved for autodidactic, self-learning instances of musical enhancement and execution. In a way, guataca is also an exclusionary term since it alludes to musical instances where musical notations are completely absent and even irrelevant, making them anti-academic and anti-classical by definition (even if the improvisational performance involves classical pieces). My recorder and I were partners.

That partnership started almost accidentally the day I turned 9. That particular day in July there were several early childhood visitors to my boarding school for the blind. As usual, when visitors came, I as the poster boy was designated as the main interlocutor among all students. That had allowed me to interact with press professionals and other kinds of visitors numerous times under diverse circumstances. But that day, the topic of my birthday came up and I was asked by one of the teaching visitors what I wanted as a gift. It so happened that we had been talking about the recorder in my music class. Hence, I asked for a recorder.

It took me years to master the recorder, with the complicit torturous experience of my family members who endured the process not without legitimate complaints. The most important feature of that learning process was the sense of freedom it allowed me. It was embodied gnosis in action. It definitely felt emancipatory. Paradoxically, nonetheless, my critical engagement with pan-disability culture^9^ (Padilla and Tan, 2019) soon taught me that both conservatory and guataca music experiences were seen among my specific disability category as a stereotypical professional sphere of allowable mastery skills. In other words, excelling in music was a naturalized attribute granted by ableist supremacist and normalizing ideologs to the disabled as something to be expected from my specific anti-ableist everyday front line interactional vantage point. This in turn created an ethical dilemma: Should I behave in ways that would perpetuate such a stereotypical self-fulfilling prophecy? Should I instead break away from the chains of its irrevocable imprisonment?

Three years ago (Padilla, 2022), I published an essay that addressed indirectly some of these issues. One of my arguments in that rather philosophical piece of critical autoethnography was that, apart from the proactive understanding and denunciation of the coloniality of power, knowledge and being, as extensively carried out by Latin American and Caribbean thinkers (see, e.g., Maldonado-Torres, 2007; Wynter, 2003), one also needs to add a fourth category: the coloniality of ethics or axiology. This involves addressing pan-disabled ethical dilemmas of the sort described above. This is not a minor dimension; it essentially entails giving up things one loves and that are part of one's identity for the sake of explicitly counteracting ableist stereotypes. In other words, for me to decide not giving it up would have involve a perfectly legitimate choice. However, it would also involve an inner struggle with one's own sense of radical exteriority (e.g., extreme othering), to use once more here Vallega's (2014) notion which he borrows from Levinas.

Looking at this epistemological struggle as a disabled scholar and activist, I now realize that my assessment of the situation at the time was probably too bound by epistemological considerations of what was demanded from me as an embodiment of disabled activism. From a gnosis standpoint, both music and positivist experiences of sociology were perfectly viable, even for a disabled person who was aspiring to an academic career in the social sciences. Adopting a much more flexible stance toward the rationalist disciplinarity of the social sciences would probably have helped as well. Perhaps many of my own anti-ableist tensions were a reflection of my colonized sense of consciousness. Such sense demanded some kind of linear externalized engagement against these stereotypical modalities of supremacist “permission” for pan-disabled people to engage in whatever activity they deem gratifying or part of their self-growth. My choice today would probably be different. Nonetheless, the damage has already been operationalized. Still, I am glad that my reflexive engagement now permits me to rescue this minor mathematics experience as a way to defend the expansive power of gnosis-based conceptions of knowledge with its consequential freedom enacting ethos as intrinsic to delinking in action.

## Final reflections on gnosis-based embodied counterstories: revolutionizing the public in public sociology?

This essay has demonstrated the reductionist and colonizing nature of epistemological, discipline-bound and rationalist conceptions of knowledge. Particularly in relation to radical cripistemologies in action (c.f., Macioce, 2022), we would like to extrapolate several important considerations regarding the use of gnosis as a reference point for equity-based transformation in delinking practices. Doing so may help take so-called public scholarship in sociology and beyond to new and transgressional horizons. We invite the reader to keep in mind nonetheless that our recurrent focus on mathematics as a deficit-based discipline colonized by the supremacist ethos of Eurocentric ideologies of normalcy is liberatory not in the sense that we desire to rescue its disciplinary integrity. Quite on the contrary, our aim through these reflections is to foster an invitational dialogue about the need to transcend disciplinary walls. We do so by embracing marginalized delinking knowledges in their own terms and in their own rights to expand on what they have done so well for centuries: disrupt established epistemological canons and normalcy-imposing monolithic domination.

In terms of decolonial delinking, both of the counterstories explored in the second half of the essay show that intersectional disability emancipation in its gnosis-based modality is relational, not merely individualist or self-enhancing. Grounded as it is in the complex realms of political society at the conflating oppressiveness of coloniality, modernity and racialized capitalism (Quijano, 1991, 2007), its emancipatory awakenings do not need to wait for policies and state sponsored formalities to be promulgated. It starts manifesting in the everydayness of anti-oppressive modes of resistance that make up cognitive and structural struggles, showing once again that, as Tuck and Yang (2012) indicated over a decade ago, the decolonial contours of actionable decolonization are not part of a metaphor, a sort of discursive game. They are living enactments of resistance and transformation that tackle, as in Paulo and Kai's embodied counterstory, the contours of embodiment level modes of coloniality in action, or in Alexis' counterstory, the colonizing dimensions of so-called independent living for disabled folks as well as their “legitimate” learning pathways outside formal/rationalist dimensions of knowledge and performativity. Furthermore, the living manifestations of decolonial disability highlighted by both of our counterstories show the false dichotomy between what some thinkers call land-based vs. epistemology-based modes of decoloniality (Motala, 2025; Zembylas, 2025). By not staying at the territorial extraction level which colonialism emphasizes, decolonial disability makes clear that the complex interactions between exploitation, oppression and knowledge-based modes of epistemicide demand a sophisticated gnosis-centered analysis which helps expose and decode the destructive mechanisms of their coloniality matrices of power (CMP). Here is where public sociology comes in. In other words, because disability dimensions seem not to have territorial implications, a narrow focus on colonialism vs. coloniality is likely to miss the power, knowledge, being/becoming, and ethical relationality contours of its complexified modes of oppression and liberation. So far, this is why most decolonial sociology has essentially ignored disability and anti-ableism matters. Ultimately, our aim with the present paper is thus to promote decolonial delinking forms of situational emancipation which can be analytically approached in several ways.

First, by realizing that in its very definition, gnosis-based, embodied counterstories turn the table on matters of expertise. One can think of Kai's reversal of teaching canons concerning delinking modes of expressivity in mathematics. Kai's relational engagement with Paulo was at once natural and transgressional. It was natural as a familial curvilinear trajectory of love and learning. Yet it became transgressional as it provided Paulo mathematical and pedagogical knowledges he could not have accessed through traditional epistemological contexts of discipline-bound conceptions of mathematics and so-called special mathematics education. This relational illustration elevates a decolonial disability justice point that underlies every section in this essay: only intersectional pan-disabled folks have the power to authentically convey their experiences in a reflexive, critically grounded manner. This realization constitutes something intrinsically contrary to epistemology as we know it, particularly within rationalist disciplines such as mathematics and mathematics education. Secondly, because of this, by radically elevating gnosis-based practices in public scholarship and activism, one can at last embody a genuine kind of marginalized scholarship and actionable decoloniality. This can be enacted within the sphere of political society, that is outside of both a political civil society and state-sponsored agendas. This in turn is at once an expression of activist research by and with pan-disabled groups as well as other intersectional actors. So far, public sociology, especially in terms of so-called inclusive equity, has been vulnerable to the enactment of expansive speaking engagements by pseudo disciplinary experts. Such experts relate to pan-disabled individuals as objects of their knowledge, not as agentic subjects who create and distribute cripistemological epiphanies as described in Paulo and Kai's counterstory. This kind of transgressive realignment of public scholarship must be carried out in terms delineated by disabled people's own experiential assessment of situations and relevant emancipatory responses (something which we are convinced is perfectly feasible and necessary for disabled groups covered by the profound and severe banner of alienation and non-agentic domination).

Our counterstories therefore reveal examples of how we can move toward liberatory mathematics education. Instead of looking for liberation within the epistemological chains imposed by disciplines, its enactment must come about through cross-coalitional cripistemologies in action. It must above all resist hegemonic knowledges with their rationalist reductionism. Our counterstories give texture to minor mathematics practices that disabled folks engage in their daily lives. Mathematics, contrary to common conception and especially in terms of how it materializes in schools, is not a rigid set of static knowledge to be ritualistically practiced. Rather, mathematics is a relational practice between and within humans and non-humans (as in Alexis' interactions with his recorder or as it is clearly manifested for blind individuals who share their lives with guide dogs. On this, see, e.g., Michalko, 1999, 2001). Such relational practice can be profoundly enhanced through these more diverse and minor ways of knowing and doing mathematics. That is to say in disrupting the very epistemic foundations of disciplinary boundaries and impositions, we create more emancipatory spaces from within and from without for genuine liberation grounded in the dignity of multiple knowledges, particularly those which have felt outside or have never been part of the canon blessed by dominant colonizing Eurocentric modes of epistemology.

## Footnotes

^1^Although it has been demonstrated that disciplinary boundaries in sociology and other social sciences are not static (e.g., Abbott and Celarent, 2017), our concern here pertains the Eurocentric nature of the epistemological basis of sociology. By this we mean the Eurocentric knowledge legacy of classic sociological thinkers such as Comte, Marx, Webber, and Durkheim, whose westernized evolutionary rationalism does not leave room for decolonial gnosis-based approaches such as those proposed in the present essay.

^2^We expand below the conceptual and methodological scope of delinking. For now, let us define it as the intentional movement away from discipline-bound knowledges, especially modalities of knowledge that epitomize rationalist and dualist ontology paradigms such as those of Descartes, Kant, and the bulk of European philosophers foundational to modernity as a colonizing and Eurocentric knowledge totality (see, Allen and Mendieta, 2021, Introduction).

^3^Like Quijano, we conceptualize coloniality as qualitatively different to colonialism, which operates at the level of extractive macro-territorial relations involving a colonizing superpower, typically a nation state. As explained in the following section, the broad relational contours of coloniality stem from colonial matrices of power (CMP) whereby coloniality/modernity/destitution get intrinsically aligned through dynamics such as those of racialized, gendered, and we would add ableist modalities of capitalism (Quijano, 2007). In this regard, our position differs from conflation-based conceptualizations of decoloniality such as that of Leonardo et al. (2023) who seem to minimize the conceptual boundaries between decoloniality, postcoloniality, and anti-coloniality, especially since they conflate colonialism and coloniality in conjunction to how they operate within educational contexts. For us, the distinctive gnosis-based kind of delinking we are espousing can only be possible in the context of decoloniality, not so much in terms of both postcolonial and anti-colonial theorizing and practice, since their ethos is tied to particular types of discipline-based epistemology.

^4^Anti-colonial thought involves both decolonial and postcolonial theorizing and practice. It also involves early decolonizing efforts aimed specifically at dismantling colonialism as Mignolo (2021, p. 17 and following) designates the specificity of this decolonizing descriptor with respect to decoloniality and decolonial delinking.

^5^Also spelled as gnoseology and gnostology; yet never to be confused with nosology, the term reserved in psychiatry for the study and classification of mental disorders.

^6^Several of Author's works expand on LatDisCrit's conceptual scope and applicability. This is the first time we employ the term Pan-Asian DisCrit. The DisCrit sub-field was initiated by Annamma et al. (2013) to argue for the intrinsic interlocking oppression interplay between race and disability as hierarchical matrices of othering. LatDisCrit merged ideas from LatCrit theory (which had originated as part of the critical legal movement of the 1970s with an emphasis on Latinx racialization and anti-discrimination concerns, see, Valdes and Bender, 2022) and DisCrit. In the case of pan-Asian DisCrit we are operating under a similar rationale, merging pan-Asian postcolonial thought with DisCrit (see, e.g., Chen, 2018; Chen et al., 2023; Coráñez Bolton, 2023). In so doing, we ground this merging process on the experiential and embodied dynamicity of firsthand counterstories whose reflexive space gives concrete metatheoretical power to crip pan-Asian alternative knowledge exploration and emancipatory learning (for an extensive treatment of emancipatory learning, see, Padilla, 2018, especially ch. 1).

^7^Figures 1.1, 1.2 and 1.3 in Mignolo (2021, ch. 1) represent graphically what is at stake conceptually and methodologically in the analysis of CMP, which Mignolo attributes to Quijano (2007).

^8^See for example Tan et al. (2019) where an expansive conception of mathematical reasoning is adopted as a way to humanize disabilities within classroom relational contexts.

^9^By this expression, we mean unique culturally relevant funds of knowledge which derive from the collective engagement in anti-ableist struggles by disabled individuals across the full spectrum of multiple categories of disability, even in cases where these forms of disability remain invisible or have not yet been embraced as part of their inner sense of identity.
